# Self-digitization chip for single-cell genotyping of cancer-related mutations

**DOI:** 10.1371/journal.pone.0196801

**Published:** 2018-05-02

**Authors:** Alison M. Thompson, Jordan L. Smith, Luke D. Monroe, Jason E. Kreutz, Thomas Schneider, Bryant S. Fujimoto, Daniel T. Chiu, Jerald P. Radich, Amy L. Paguirigan

**Affiliations:** 1 Division of Clinical Research, Fred Hutchinson Cancer Research Center, Seattle, Washington, United States of America; 2 Department of Chemistry, University of Washington, Seattle, Washington, United States of America; Queen's University Belfast, UNITED KINGDOM

## Abstract

Cancer is a heterogeneous disease, and patient-level genetic assessments can guide therapy choice and impact prognosis. However, little is known about the impact of genetic variability within a tumor, intratumoral heterogeneity (ITH), on disease progression or outcome. Current approaches using bulk tumor specimens can suggest the presence of ITH, but only single-cell genetic methods have the resolution to describe the underlying clonal structures themselves. Current techniques tend to be labor and resource intensive and challenging to characterize with respect to sources of biological and technical variability. We have developed a platform using a microfluidic self-digitization chip to partition cells in stationary volumes for cell imaging and allele-specific PCR. Genotyping data from only confirmed single-cell volumes is obtained and subject to a variety of relevant quality control assessments such as allele dropout, false positive, and false negative rates. We demonstrate single-cell genotyping of the *NPM1* type A mutation, an important prognostic indicator in acute myeloid leukemia, on single cells of the cell line OCI-AML3, describing a more complex zygosity distribution than would be predicted via bulk analysis.

## Introduction

Clonal evolution in cancer—the selection for and emergence of increasingly malignant clones during progression and therapy—has been highlighted as an important phenomenon in the biology of leukemia and other cancers.[1–9] While emerging evidence shows that tumors can be composed of multiple cancer cell clones, it is unclear how the relative fitness imparted by genetic variation between individual cells (clonality) contributes to the evolutionary race between these cancer cell clones. Expanded access to next generation sequencing (NGS) platforms, as well as sensitivity improvements, have broadened our understanding of intratumoral heterogeneity (ITH), as we are now able to describe genetic variability within diseases.[10] However, our ability to deduce the clonal composition of individual patient samples has been limited by the use of DNA sequencing data from bulk tumor samples. Current methods typically rely upon the extrapolation from mutant variant allele frequencies (VAF) of DNA from the metagenome of bulk tumor samples. This approach requires a set of assumptions about the possible zygosity distribution among the single cells that comprise the admixture tumor sample. The basic evolutionary framework into which these assumptions fit is the idea of a linear sequence of clonal expansions in which each mutation occurs only once, driving a new clonal expansion in which all further mutational events occur heterozygously. While studies have demonstrated these “linear” models of clonal evolution in certain cases, with new clones and sub-clones clearly arising serially, complex “branching” tree models have also been described, contributing to evidence of convergent evolution.[11,12]

**Fig 1:**
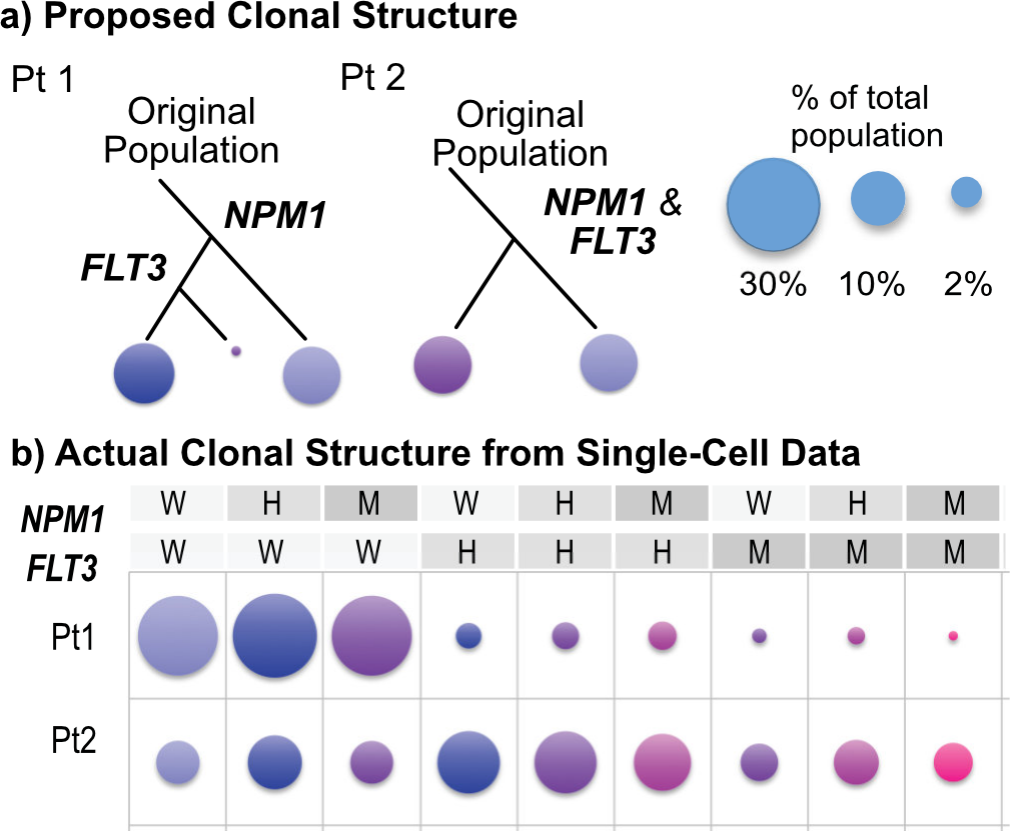
Genetically distinct clonal population frequencies in AML with respect to *FLT3*-ITD and *NPM1*. (**a**) Using the assumptions of heterozygosity and serial clonal expansions, bulk data for patients 1 and 2 from our previous study would suggest fairly simple clonal structures with either 2 or 3 clonal populations (circle area is proportional to the contribution to the total population of that clone). (**b**) However, when analyzing these patient samples using single-cell genotyping for these two loci, clonal distributions of *NPM1* and *FLT3*-ITD insertions are very different than the predicted structure (W: wild type, H: heterozygous, or M: homozygous mutant for the locus, bubble area is proportional to the percentage of cells in the total population of analyzed cells with the indicated joint *NPM1/FLT3*-ITD genotype). Via our previous approach to targeted single cell genotyping via macro-scale fragment analysis, we demonstrated with high statistical confidence that all possible zygosity combinations occur, though at variable frequencies.

Acute myeloid leukemia (AML) has previously been considered a clonal disease, assuming a common genetic precursor cell, though it has been more recently understood that clonal relationships in AML can be far more complex.[13] When loci exist with ITH structures or evolutionary patterns that do not match the bioinformatic model employed, we will inevitably describe an incorrect, and typically oversimplified, clonal structure.[10] We have previously published evidence of this complex clonality in AML using a targeted single-cell genotyping approach focused on common mutations that correlate with outcome.[14–17] Based on bulk patient sample data, the predicted clonal structures with respect to these two mutations for patients 1 and 2 in our cohort are shown in (Fig 1A), however, the underlying clonal structures determined via single-cell targeted genotyping were significantly more complex than predicted (Fig 1B).[10] This study used macro-scale PCR reactions and capillary electrophoresis to identify zygosities of individual cells. Though this study was critical to clarifying our understanding of ITH, the approach proved infeasible to apply in a clinical context as an assay for AML ITH assessment. The results from this study suggested that a targeted, single-cell genotyping approach with higher throughput of cells per sample, lower cost per cell, and reduced process complexity would be ideal for future integration of this type of targeted approach into clinical laboratories.

**Fig 2:**
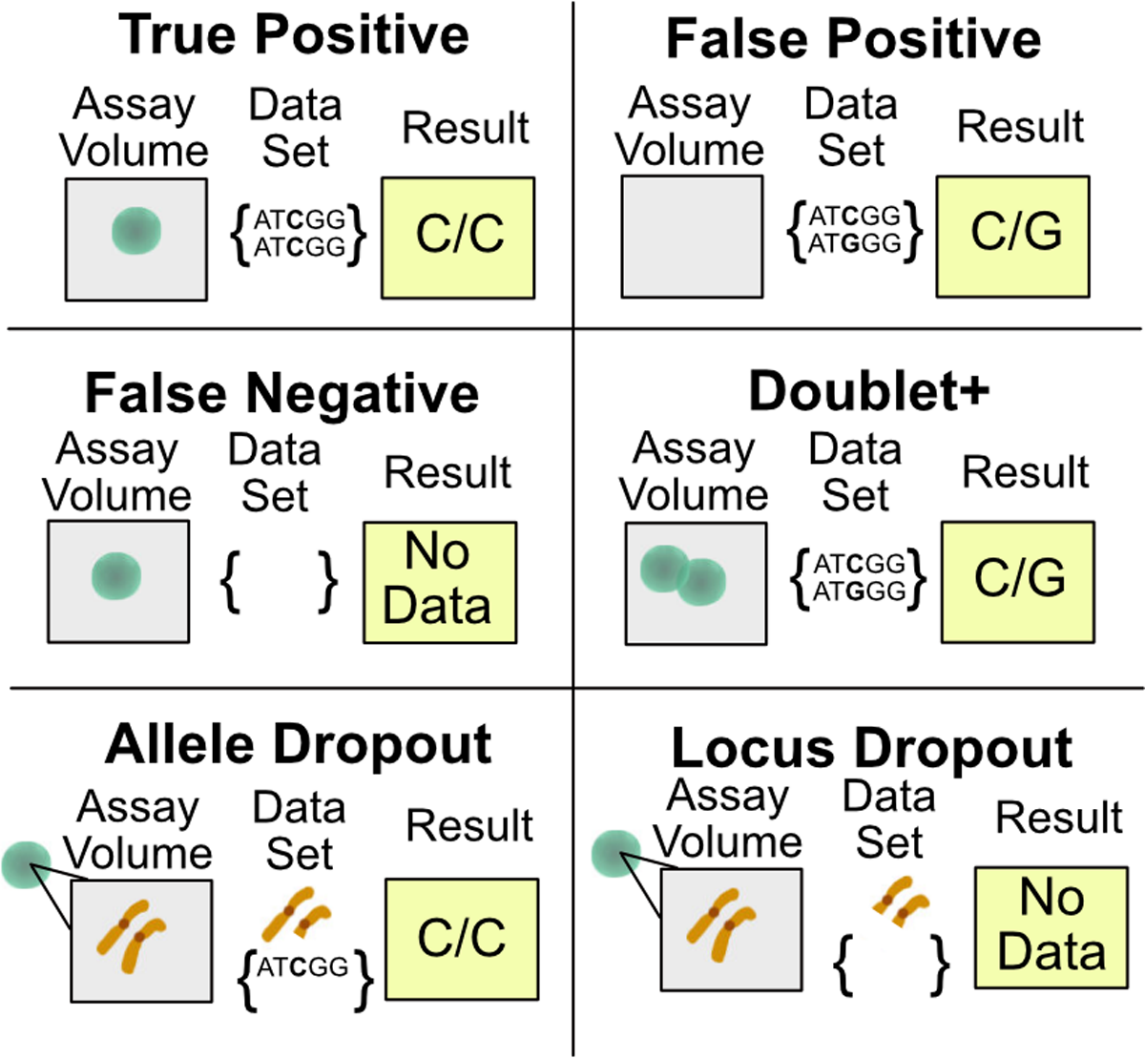
Single-cell analysis quality control statistic types. Single-cell sequencing or genotyping platforms are subject to multiple types of errors that are far more challenging to address than more traditional, bulk approaches. Single-cell assays thus require significant, specialized validation with these issues in mind. The general categories into which these statistics fall include: true positive (accurate data from one cell), false positive (data is erroneously obtained from an assay volume that does not contain a cell), false negative (a cell is present, but data is not obtained), doublet+ rate (more than one cell is present in an assay volume, but data is indistinguishable from that obtained from a single cell), allele dropout (an allele is present in an assay volume yet erroneously not detected in the data), and finally locus dropout (failure of a genetic locus to be represented in the dataset from a given cell). The impact of the failure rates in particular on the interpretation of the resulting single-cell data can be significant when describing the clonal composition in an unknown human specimen.

The field of single-cell genetics promises to tweeze apart the heterogeneity we now appreciate in bulk samples, but this work is still in its infancy with respect to its relevance to understanding human disease. NGS based DNA sequencing of single cells, while providing some degree of refinement of clonal distributions based on bulk data, is still subject to challenging technical and statistical issues common to all single-cell analyses. A detailed description of the statistics and failure modes of various approaches to single-cell sequencing and genotyping have been reviewed in detail by Wang and Navin,[18,19] which highlights the unique challenges to even identifying how to approach the single-cell assay validation process itself. While the particular failure rates of single-cell assays tend to be very platform- and molecular biology workflow-specific, the main types of single-cell assay quality control (QC) statistics fall into the six categories described in Fig 2. While a variety of approaches for single-cell analysis have been demonstrated, most have not completely addressed the unique statistical challenges of single-cell assays nor is the approach for validating them straightforward for potential users of the technologies.

Many single-cell genomic technologies aim to provide wide genome coverage (whole genome, whole exome, or large panels of loci), however, in some instances it is preferred to perform a targeted analysis on a small number of loci if the trade-off is for higher data quality. Panel-based sequencing approaches are designed to generate data from a defined region of the genome from single cells to identify concurrent mutations, but tend to suffer from high rates of locus dropout, and it is infeasible to determine the allele dropout rate for all the loci in the panel.[20–25] Thus they require post-processing of the results, such as assuming allele dropout to be balanced for each allele and the same rate for all loci (which is unlikely to be universally true), and using consensus calling for cell genotypes (for example requiring at least 3–5 cells to have the same genotype in order for it to be identified as a valid clone). Beyond statistical limitations, these high-throughput single-cell genetics approaches can have a high cost per cell and long bioinformatics lead times, affecting their utility. In contrast, targeted approaches can allow for increased quality statistical descriptions, including allele dropout rate measurement for every locus, and can be performed more quickly and at lower cost, but at the expense of the ability to identify co-occurrence of mutations.[10]

Given these relative strengths, the choice of panel-based or targeted single-cell genomic approaches depends on whether there are recurrent mutations in the disease that can be directly targeted, or if the genetics are far more variable and the single-cell analysis approach is mainly exploratory. In AML, most patients do not appear to have large numbers of mutations in their leukemia that are recurrent in the population, thus ultimately describing the intratumoral clonal distribution will require targeted, patient-specific genomic approaches. Thus in cancers with limited numbers of mutations per tumor, using bulk sequencing data to identify specific loci of interest, followed by single-target assays that produce less genome coverage but at higher confidence, is an appealing approach for hypothesis-driven ITH studies for many cancers.

In order to perform single-cell genotyping in a way that simultaneously produces relevant statistics but also provides us an understanding of a patient’s intratumoral clonal distribution, we have developed a targeted single-cell genotyping platform using the microfluidic self-digitization chip (SD chip). This device allows us to perform both cell imaging and genotyping in stationary, nanoliter (nL)-scale volumes, generating data that provide additional descriptions of uncertainty as well as identify the population zygosity distribution. Many of the genetic assessments of ITH performed on bulk cancer specimens will require the final description of ITH to be informed by single-cell data rather than solely relying on computational deconvolution of the bulk sequencing data to describe the clonal structure. Targeted single-cell genetic analysis tools such as those presented here can be used to test ITH hypotheses with statistics describing technical and biological variability. This will extend our ability to assess ITH beyond only descriptive data collection methods.

## Results and discussion

### SD-genotyping chip device design and molecular workflow

**Fig 3:**
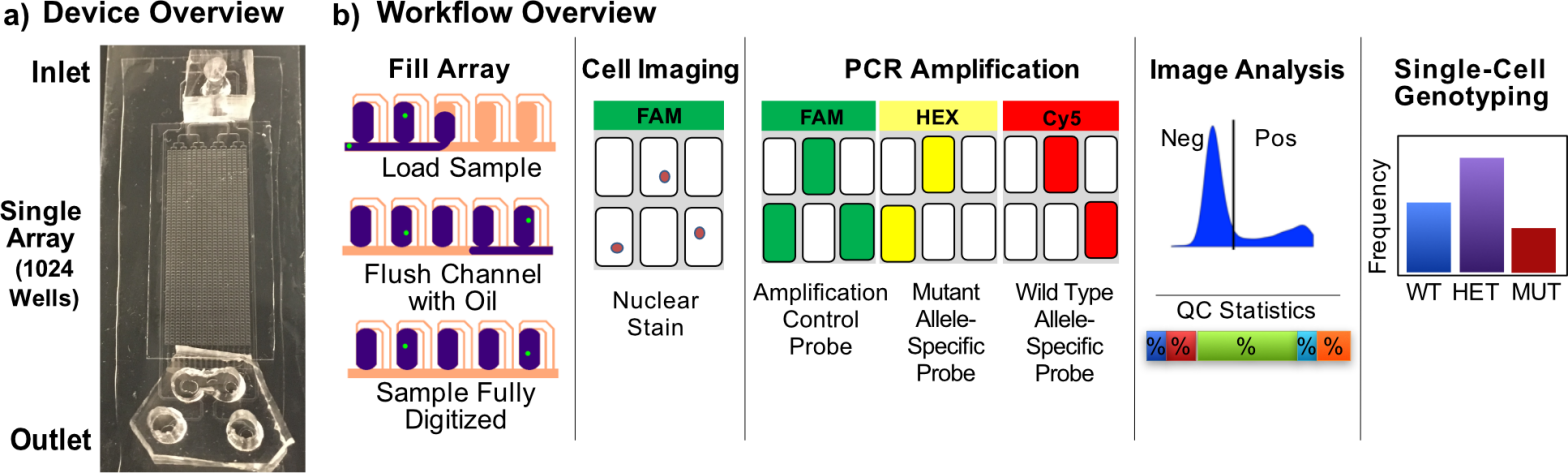
Overview of the single-cell SD genotyping chip workflow. (**a**) The chip is composed of PDMS bonded to a microscope slide with a bonded glass coverslip over the array and surrounding oil channel to prevent sample evaporation. (**b**) Cell-PCR mix suspension is pipetted directly into the inlet reservoir flows into the wells of the array by applying vacuum to the outlet port. Once the sample volume is loaded into the wells, the main channel is flushed with oil to fully digitize each 8nL volume. The arrays are imaged to identify wells containing single (or more) cells. PCR is performed in the digitized volumes by thermalcycling of the entire chip, and fluorescence is quantified at endpoint in three fluorescence channels (FAM for the amplification probe, HEX for the mutant allele-specific probe and Cy5 for the wild-type-specific probe). In wells containing a single-cell and with amplification probe fluorescence above a set threshold, as well as allele-specific probe fluorescence above their respective thresholds, we can generate QC statistics for the array and determine zygosities of single cells.

The structure and characterization of the SD chip has been described previously, and this device has been used for digital PCR detection of both mRNA and DNA.[26,27] The SD chip device mechanism and workflow for single-cell genotyping are illustrated in Fig 3. Our assay for single-cell genotyping for the *NPM1* mutation consisted of a single set of PCR primers specific for the region in *NPM1* where the mutation occurs, a low-concentration intercalating dye to image single nuclei, and three hydrolysis probes (one amplification control probe specific to the gene, and two specific to either the wild-type or mutant allele). Imaging of the digitized volumes before PCR allowed us to identify the specific well locations of single cells, empty wells, or wells containing more than one cell (doublet+). Post-PCR imaging of the device using a laser scanner was used to identify PCR. A single-cell genotype was only reported when the presence of a single cell was confirmed in a filled well, amplification probe signal was detected, and at least one of the wild-type or mutant probe signals were detected. Analyses of cell and post-PCR images of the arrays in concert allowed us to not only determine the *NPM1* genotype of single cells, but also to calculate error rates for every array, including false-negative, false-positive rates and doublet+ rates, which are typically challenging or impossible to generate from existing single-cell analysis approaches.

### Single-cell genotyping molecular assay development

The *NPM1* type A mutation is an insertion mutation consisting of a four base-pair repeat. Allele-specific hydrolysis probes specific to the wild-type or mutant allele containing locked nucleic acids around the insertion site were designed and validated. A third hydrolysis probe located within the amplicon but outside the region of the mutation served as an amplification control to verify successful amplification of the *NPM1* locus in a given cell, allowing for evaluation of QC statistics independent of the zygosity of the individual cell. Plasmids with inserts containing the amplicon with the wild-type or mutant allele as well as wild-type or heterozygous cell line DNA were used to confirm the specificity of the probe designs in bulk PCR.

To extend our assessment of specificity for single-cell genotyping, we validated the three-probe suite in bulk PCR conditions that mimicked the conditions seen by cells in wells of the SD chip. We used concentrations of viable cells input directly into the PCR reaction volume to give an effective cell concentration commensurate with a single cell in an 8 nL volume as seen on-chip. Both wild-type cells (KG1a) and a cell line with the *NPM1* mutation (OCI-AML3) (bulk VAF_NPM1_ of 50%) were used in validation. All reactions in bulk were run with the thermal profiles used in the chip, which required using slower ramp rates and a modified hot start.[28] We confirmed that the target amplicon was produced under these conditions (S1 Fig). The bulk model PCR readout was the total PCR endpoint fluorescence of wells with input sample, compared to that of the no template control wells, or the probe fluorescence differential.

We optimized probe concentration, nuclear stain concentration, salt concentrations, and surfactants to maximize the endpoint probe fluorescence differential and maintain specificity in this bulk model. A low concentration of the DNA intercalator EvaGreen (0.5x) was used for imaging cells prior to PCR; this was found to have no impact on the specificity of allele-specific probes (S2 Fig). Increasing PCR buffer to 1.5X and MgCl_2_ concentration to 3 mM contributed to improved fluorescence differentials from plasmids, DNA, and cells. We observed no noticeable change in specificity or endpoint fluorescence at various concentrations of the surfactant Triton X-100 (S3 Fig). Cell imaging in PCR buffer was performed to verify that the buffer or surfactant additives did not cause premature lysis of the nucleus which would contribute to false positive amplification (S4 Fig). For single-cell experiments in the SD chip, we found that PCR buffer with added 0.02% Triton X-100 minimized false positive and false negative rates compared to other buffer conditions tested (S5 and S6 Figs).

### Image processing and analysis

We evaluated on-chip PCR results from control templates from multiple replicates to determine appropriate fluorescence thresholds, to reduce potential bias in the evaluation of QC statistics and zygosities from single cells. Details regarding the imaging processes and data reduction are described in the Materials and Methods section and S7 Fig. A self-contained example containing all the raw data and scripts required to process the raw data and generate plots included in the manuscript is available via GitHub (see Methods). After image processing, the resulting data from individual wells in an array is the integrated fluorescence intensity normalized to the well area (adjusted integrated density, or AID). The distributions of these values for the three fluorescence channels are analyzed to determine which wells are considered positive or negative. In order to determine platform-specific thresholds, we first analyzed no template control replicates (N = 2) to determine the expected distribution of AID of PCR-negative wells in the amplification probe channel. We found that when a threshold was set at three standard deviations above the mean, all well AID’s of the no template control replicate arrays fell below the threshold. A similar analysis was performed, using wild-type or mutant plasmids as the template material, to set a threshold value for the two allele-specific probes. Due to differences in the fluorescence signal distribution for each fluorescent probe in on-chip wells, ideal thresholds were six standard deviations above the mean for the mutant-allele probe and five standard deviations above the mean for the wild-type allele probe. We calculated a false allele rate, defined as the number of false alleles (mutant calls in an array of wild-type plasmid template, or wild-type calls in an array of mutant plasmid template) divided by the total data points from the replicates. We found a mutant false allele rate of 0.3% (2 false mutant alleles out of N = 610 wild-type plasmid data points) and a wild-type false allele rate of 0.2% (2 false wild-type alleles out of N = 885 mutant plasmid data points). From this, we concluded that these thresholds were sufficient to assign PCR positivity while minimizing false-positive allele calls (additional detail in Methods).

### Allele dropout validation

Allele dropout (ADO) is a phenomenon which occurs in many PCR methods, but is uniquely challenging to identify and mitigate in the context of single-cell analysis. For the SD-chip, we address the issue of allele dropout by employing a heterozygous plasmid template to assess the frequency at which amplification occurs but one allele is not detected. Bias occurs in genotyping results when the ADO rate is not balanced between two alleles at a locus, or uniformly distributed across all loci.

To perform analysis of ADO on the SD-chip, the input concentration of heterozygous plasmids was adjusted to maximize the likelihood that amplification-positive digitized volumes would have single-plasmid occupancy (and thus only two copies of the amplicon). Data from heterozygous plasmids from 6 individual array replicates, on two separate days (run from different reagent mixes on different days) was combined with Poisson statistics (described in detail in Methods) to generate a most conservative estimate of the number of allele-dropout events for each of the two alleles. The ADO rates observed were 5.4% ± 2.3 for the wild-type allele (events where heterozygous plasmids were genotyped as mutant), and 8.5% ± 2.1 for the mutant allele (error is standard deviation between 6 array replicates with total zygosity calls included of 1095). Similar ADO rates were observed when using allele-specific probes with the specific fluorophores conjugated to them swapped (details in S1 Text). These rates are consistent with those previously calculated using limiting dilution of heterozygous *NPM1* plasmids in 384-well plate using fragment analysis as the readout for this same locus,[10] and are much lower than other published single-cell assays.[19]

### Single-cell quality control statistics

**Fig 4:**
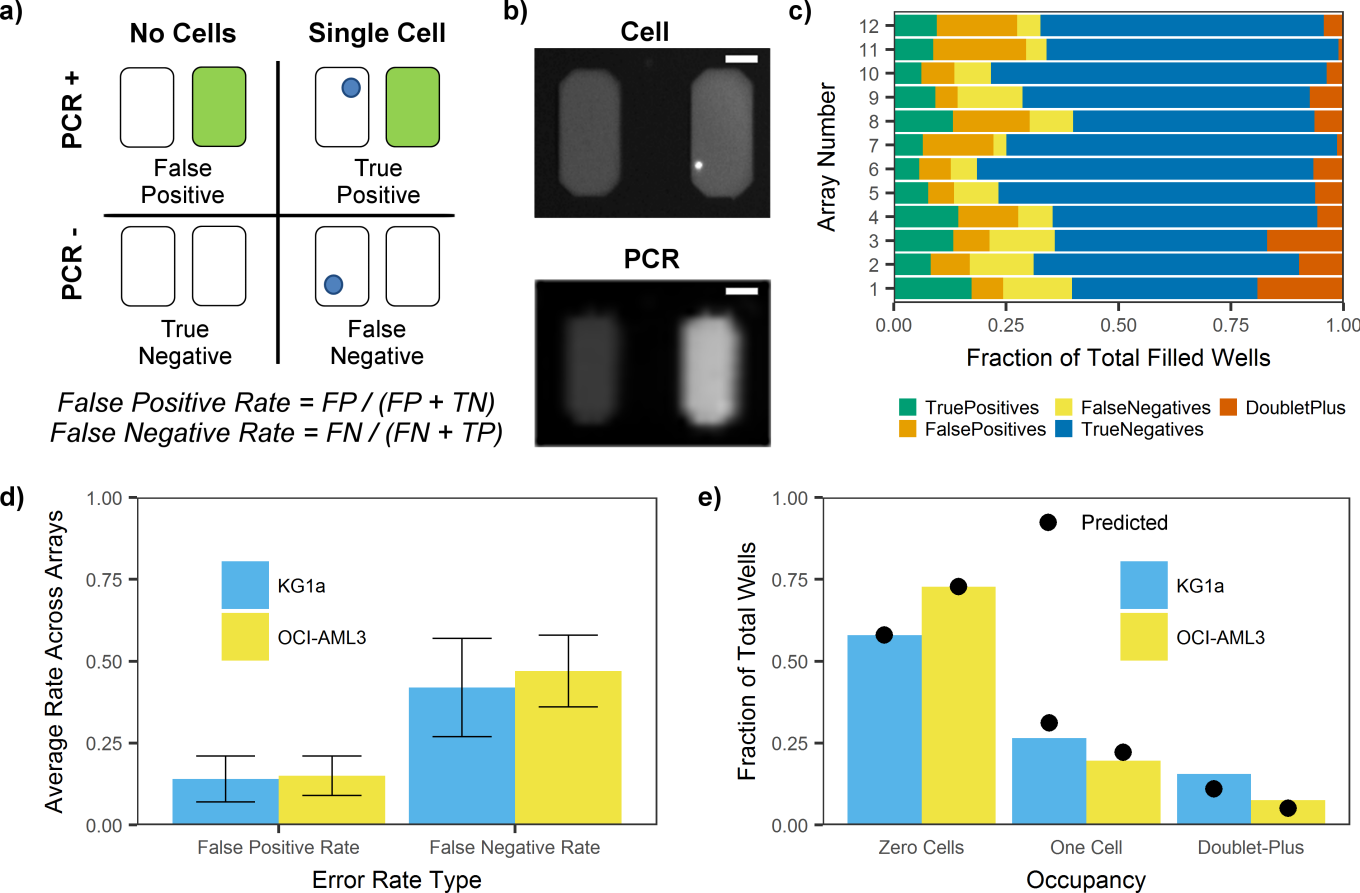
Method quality statistics. (**a**) Based on cell and endpoint PCR imaging, wells can be categorized as true positive (TP), false positive (FP), false negative (FN), and true negative (TN). These counts allow us to calculate a false positive and false negative rate for each array to assess performance. (**b**) Images show two wells of the SD chip before (top) and after (bottom) PCR. A single OCI-AML3 cell was identified in the right well, and the well was PCR positive after thermalcycling. The scale bar is 100 μm. (**c**) For the 12 SD chip arrays used for genotyping single OCI-AML3 cells, colored bars represent the fraction of filled wells that fall into each category described in panel A. Wells with more than one cell (doublet+) are also reported. (**d**) False positive rates and false negative rates were calculated for each OCI-AML3 or KG1a single-cell genotyping SD chip array. The average rate between arrays is reported for arrays with KG1a cells (blue) and arrays with OCI-AML3 cells (yellow). Scale bar represents standard deviation (N = 12 arrays with OCI-AML3 cells, N = 4 arrays with KG1a cells). (**e**) The fraction of wells containing no cells, one cell, or more than one cell (doublet+) was quantified from images of cells in arrays of the SD chip. Using Poisson’s equation, we calculated a predicted number of wells (black circle) that would be expected to contain one cell or more than one cell using the number of wells containing zero cells. For arrays containing either KG1a cells (blue) or OCI-AML3 cells (yellow), the actual number of wells with one cell was below the predicted number while the number of multiple cell wells was higher.

An inability to quantify uncertainty in single-cell assays has historically been a challenging aspect to validating and interpreting the data produced by these assays. Due to the fundamental limitations of single-cell analysis (i.e., one can only assay an individual cell once, replicates are challenging, nucleic acid amplification is always required), generating single-cell specific QC statistics requires validation of both the platform itself as well as run-specific metrics. With combined cell imaging and allele-specific PCR results, we are able to identify wells that do not meet minimum quality criteria (Fig 4). After image analysis and filtering of un-filled wells (<50% area), wells are categorized into four types, true positive (TP), false positive (FP), false negative (FN) or true negative (TN), (Fig 4A). As our method measures a single locus, a false negative event is equivalent to a locus dropout event. We can then calculate a variety of QC statistics such as the false negative rate (FNR) and the false positive rate (FPR), as proportions of relevant wells across arrays analyzing OCI-AML3 or KG1a cells (Fig 4). False positives, which could be due to premature lysis of cell nuclei or incomplete cell staining, and false negatives, which could come from failure to amplify or false identification of single cells, might be improved with further optimization of surfactant additives and thermalcycling protocols.

We have observed during this validation that when cells are manipulated in buffer mixes suitable for molecular biological assessments, a wide range of degrees of cell lysis occurs simply by exposure to the reagents necessary. The possibility of cell lysis prior to digitization and PCR amplification is a very context-dependent phenomena and an important source of possible PCR contamination and false positives in single-cell datasets. Because all single-cell analysis techniques require a significant amplification of genetic material, not surprisingly these assays are also uniquely sensitive to contamination beyond what is typical for NGS and other molecular assays, making the rate of false positives in single-cell methods especially important.

For single-cell analysis systems without imaging capabilities, the assumption of a random distribution of cells in the sample volume is used to estimate the number of doublet data points in a sample. To test the randomness of cell isolation in our system, we generated an estimate of expected cell occupancy counts using the Poisson distribution from 1649 OCI-AML3 cells and 797 KG1a cells across all arrays containing cells (12 arrays of OCI-AML3 cells, 4 arrays of KG1a cells). As shown in Fig 4D, even for this suspension cell line, we found that the number of doublet+ containing wells was consistently higher than that predicted from the Poisson density function. Possible causes for this include cell-cell adhesion or filtering effects from defects in microstructures. These results indicate that for methods that do not use imaging, but instead assume a Poisson distribution of cells, the rate of doublet+ measurements could be underestimated.

### Single-cell genotyping

**Fig 5:**
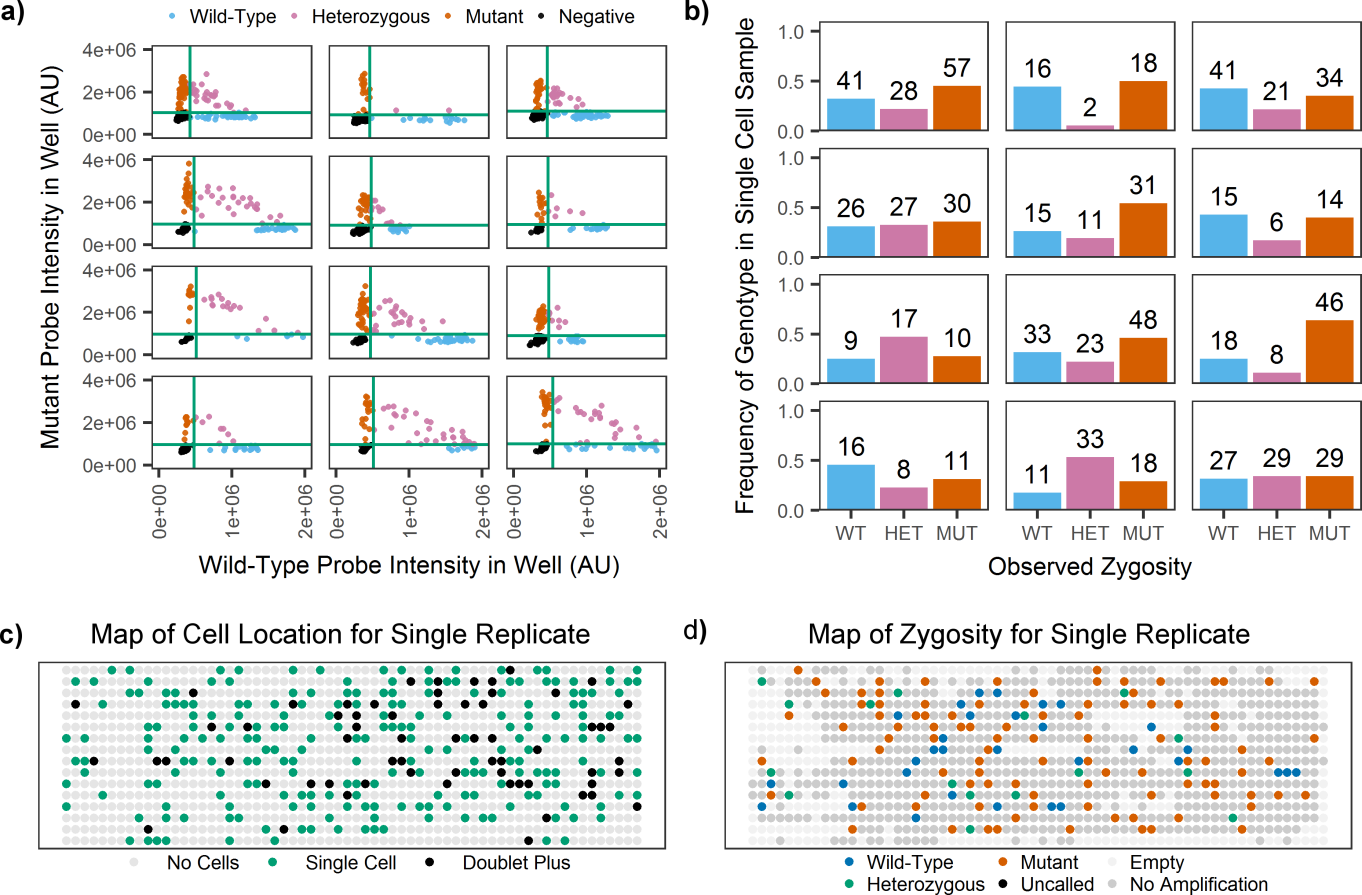
Single-cell genotyping results. (**a**) Scatterplots for each SD chip replicate containing OCI-AML3 cells are shown as 12 panels. The fluorescence intensity in the mutant probe channel is plotted vs. the wild-type channel intensity for single-cell containing wells. Thresholds in each channel (green) separate single-cell wells into mutant, heterozygous, wild-type, or false negative (FN). (**b**) For the same twelve SD chip replicates, bar plots show the frequency of single cells in the population that were assigned as wild-type, heterozygous, or mutant. (**c**) A representative map of wells in a single replicate shows the results from cell imaging for each well. Each dot represents one well of the device, which are categorized as having no cells, a single cell, or multiple cells (doublet+). (**d**) For the same array, a map of zygosity shows the location of wells categorized as wild-type, heterozygous, mutant, empty of aqueous solution (Empty), no amplification probe fluorescence (NonAmp), or having positive amplification probe fluorescence but negative allele-specific probe fluorescence (UNCALLED).

**Fig 6:**
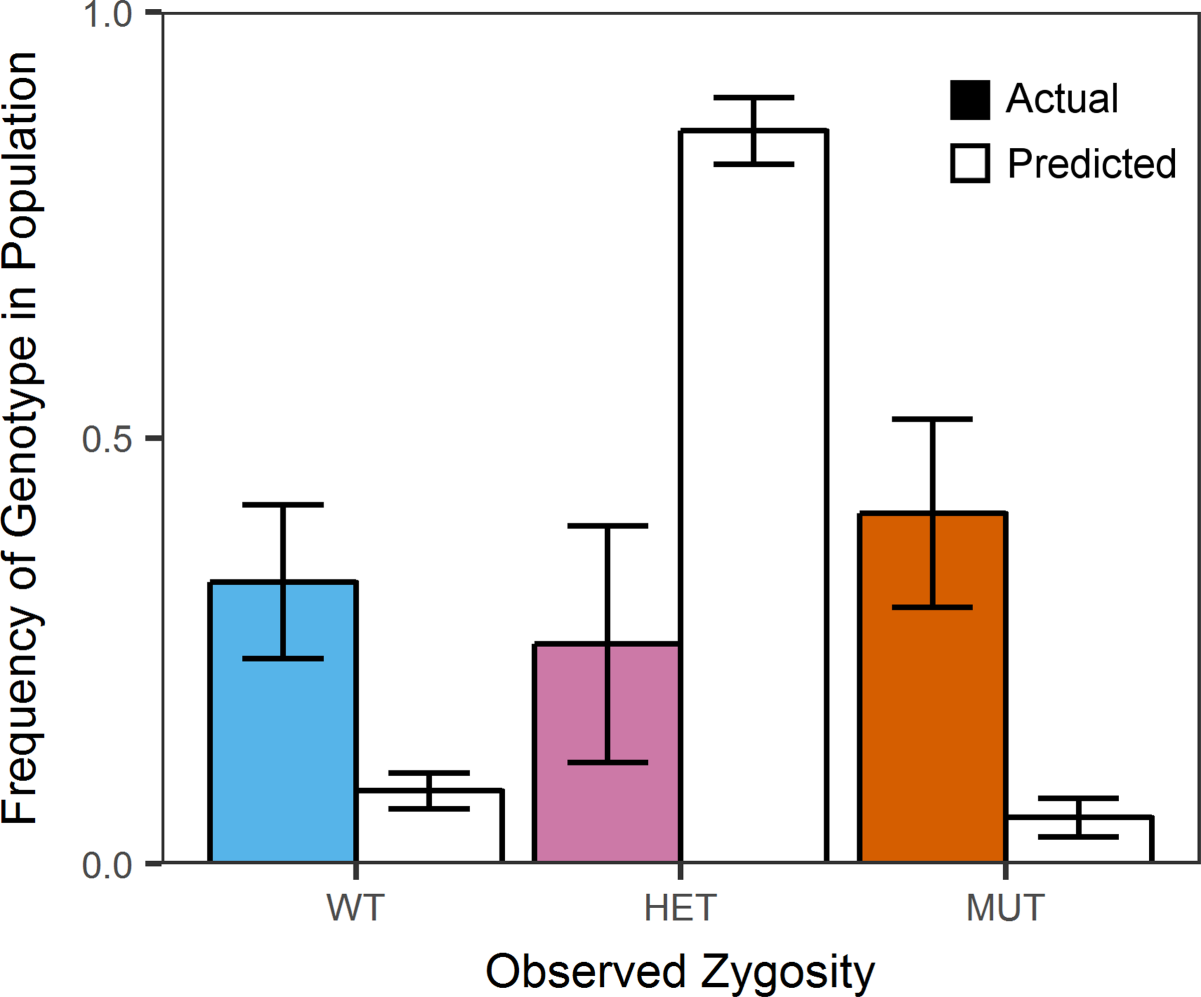
Mean actual observed zygosity of OCI-AML3 cells compared to zygosity predicted from variant allele frequency. Colored bars represent the mean zygosity frequency measured in single cells (847 single-cell measurements total). White bars with black outline represent the predicted mean zygosity frequency we expect our method to report for a sample for which all single cells are heterozygous. Actual genotype data is for N = 12 arrays of OCI-AML3 cells. Predicted genotype data is from measurements of single plasmids with one copy of each wild-type and mutant allele. Predicted genotype data are for N = 6 arrays of these heterozygous plasmids. In each case, genotype frequencies were quantified in each array; reported means were calculated between arrays. Error bars represent standard deviations of these measurements.

We determined genotypes of single OCI-AML3 and KG1a cells based on wells where we could confirm the presence of a single cell, and could identify as positive for the amplification probe and at least one of the allele-specific probes. Genotyping results indicated that all 467 KG1a cells were wild-type as expected; results from 847 OCI-AML3 show a mix of wild-type, mutant, and heterozygous cells. Fig 5A and 5B shows the genotyping results from the 12 replicates, and Fig 5C and 5D shows a representative map of the well-specific results for a single replicate. Using this single-cell data from OCI-AML3 cells to calculate a VAF for the bulk sample, as VAF = (1N_Mutant_ + 1/2N_Heterozygous_ + 0N_Wild-type_)/N_Total_, where N is the count of single cells for each genotype, we arrive at a VAF of 54% ± 7 (mean +/- standard deviation across 12 replicates), which is consistent with the bulk VAF (50%) we have observed in this cell line. If we follow typical assumptions for using VAF to determine clonality, a bulk VAF of 50% corresponds to a single population of cells that are all heterozygous mutant. Single-cell genotyping in the SD chip revealed that the population distribution of OCI-AML3 cells was significantly different from that expected based on this bulk model, even when accounting for the known rates of allele dropout (p-value < 1e-5), as shown in Fig 6. We have previously observed this deviation from a homogeneous heterozygous population via another approach, thus regardless of the method employed, we can confirm that the bulk assumption of heterozygosity is not accurate for this cell line.[10]

## Conclusions

We have developed the SD genotyping platform which employs an SD chip and allele-specific PCR “suites” of locked nucleic acid (LNA), multiplexed, hydrolysis-probes to simultaneously and specifically detect locus amplification and both wild-type and mutant alleles. Alongside these locus specific assay “suites,” we have identified the critical types of reference materials and controls for validating new single-cell assays. A critical strength of the SD chip genotyping approach is the ability to image the cells prior to PCR and retain their physical location information throughout the data analysis. Imaging allows us to address multiple aspects of uncertainty that have previously been left unaddressed in nearly all single-cell genetic analysis techniques. Our technique allows for a straightforward approach to identification of false positives as well as multi-cell wells in such a way that when these artifacts occur, we can identify them and remove them from the analysis.

While bulk approaches are providing much needed insight into the inter-patient variability present in cancer, it will be crucial to, in parallel, foster the development of approaches to refine our understanding of the underlying intra-patient heterogeneity.[10,29,30] The ability to track clonal diversity over the course of therapy would allow us to determine what impact clonal dynamics have on outcomes and would be a useful tool in designing therapies informed by the possibility of clonal evolution. One could apply clonal deconvolution models to bulk DNA sequencing data to propose clonal structures and identify variants for which the zygosity distribution in the population is predicted to be most relevant to therapy. Single-cell targeted genotyping could then be used as a hypothesis testing tool to further refine our understanding of bulk DNA sequencing deconvolution and zygosity distributions of loci of interest to leukemia and cancer more broadly. The integration of this technique downstream of bulk analysis will allow us to analyze up to three loci per run for a given patient sample. This three-array chip design could be scaled up as needed when more than three loci are mutated in a given patient specimen. By using the single-cell data to reduce or confirm complexity in predictive models rather than to “discover” it, we could employ hypothesis testing using single-cell population statistics to address the unavoidable range of artifacts in any single-cell analysis that make resolving technical and biological variability challenging. The combination of larger scale sequencing efforts and targeted, refining single-cell technologies will allow us to better integrate conclusions from ITH studies into a clinically actionable understanding of how cancer evolves.

## Materials and methods

### Cell lines and template reference materials

OCI-AML3 cells (DSMZ) and KG1a cells (ATCC) were cultured in 20% FB Essence (VWR Life Science) in RPMI media (Gibco). Genomic DNA was extracted from cell line lysates using Qiagen Gentra PureGene Cell Kit (Cat No./ID: 158745), according to the manufacturer’s protocol. Reference sequence plasmids for *NPM1* alleles were lab-designed and purchased from Life Technologies. Single-zygosity plasmids (homozygous mutant or homozygous wild-type) contained one copy of the amplicon region per plasmid. Heterozygous plasmids contained one copy of wild-type and one copy of mutant amplicon region inserted in series. All plasmids were sequence-validated by Life Technologies and in house. OCI-AML3 cells and all mutation-containing plasmids had the “Type A” *NPM1* insertion, which is the TCTG tetranucleotide duplication in position c.860_863dupTCTG (NM_002520.6), the sequence of the insertion that is present in ~80% of AML patients positive for the *NPM1* insertion.

### Gene and allele discrimination primers and probes

Primers were designed to amplify a 162 base-pair (166 bp with insertion) stretch of genomic DNA spanning the location of the *NPM1* 4-bp insertion. Locked nucleic acid (LNA) allelic discrimination hydrolysis probes were designed with guidance from You et al.,[31] though the specific detection of an indel is not specifically described. The probe designs were validated and optimized for application to single-cell genotyping and their ability to specifically detect the mutant and wild-type alleles prior to application on chip. LNA-containing probes were single-quenched fluorescent probes with non-fluorescent quenchers purchased from IDT (Integrated DNA Technologies). A FAM-labeled hydrolysis probe was designed outside the insertion site as a positive control to confirm amplified product. Cy5-labelled wild-type-specific and HEX-labelled *NPM1*-Type A insertion-specific probes were designed for genotyping. The same probe sequences for the allele-specific probes were also labeled with the alternate fluorophore (i.e., wild-type probe with HEX and mutant specific probe with Cy5) to evaluate the impact of fluorescence channel specific bias as opposed to allele-specific bias. Fluorophores for each probe were chosen to minimize spectral overlap and for optical compatibility with the Biorad CFX PCR system, our Olympus microscope filter set, as well as the available channels on our Typhoon Trio. Primer and probe sequences are listed in S1 Table.

### PCR conditions and sample preparation

PCR reagents were purchased from Invitrogen except where indicated. Primers and probes were ordered from IDT. Optimized reagent concentrations in the PCR were as follows: 1.5x PCR Buffer (from 10X PCR buffer), 5 mM MgCl_2_, 600 nM each dNTP, 750 nM each primer, 600 nM amplification control probe, 600 nM wild-type probe, 600 nM mutant probe, 0.1% BSA (Sigma part no. 10711454001), 0.5x EvaGreen (Biotium 20X EvaGreen), 0.02% Triton X-100 (Sigma part no. T8787), and 3 U/10 μL Platinum Taq. For each reaction, 2μL of template (cells, plasmid, or PBS) was added for 10μL reactions in bulk PCR. Cultured cells were centrifuged at 200 rcf for 3 minutes and resuspended in PBS (Thermo Fisher no. 10010023) to a concentration of 1000 cells per μL. PCR was run on a CFX384 Touch Real-Time PCR Detection System (BioRad). Typical PCR hot start protocols were modified to enhance cell lysis and began with 3 cycles of 3 minutes at 95°C and 1 minute at 60°C, followed by 40 cycles of 15 seconds at 95°C and 45 seconds at 60°C (S1 Fig). Ramp rates were adjusted to 1.5°C/s increasing and 0.9°C/s decreasing to match the inherently slower ramp rates experienced when using the thermal cycler flat plate adapter for SD chip samples.

SD chip PCR reagent, cell, and plasmid concentrations were the same as for bulk PCR. Samples were prepared by adding 4μL of template (cells, plasmid, or PBS) per 20μL of PCR buffer, and then transferring 15μL of this sample to inlet reservoir of the device. Filled SD chips were cycled on an ABI 2720 Thermal Cycler (Thermo Fisher) fitted with a Techne *in situ* Hybridisation Adapter (Fisher Scientific part no 13245153). A thin film of light mineral oil (Sigma part no. M8410) was placed between the device and the plate to improve contact during cycling. The thermal cycling profile for cycling SD chips was adjusted such that measured temperatures between the plate and device where as intended. Programmed cycling conditions were 3 cycles of 3 minutes at 95°C and 1 minute at 60°C, followed by 40 cycles of 15 seconds at 97°C, 5 seconds at 50°C and 45 seconds at 60°C.

### SD chip fabrication and loading

Details of SD chip fabrication have been described previously.[32] The desired micro-scale features were drafted using CAD software (AutoCAD) and printed in high-resolution on transparent film (Fineline Imaging). Masters were constructed from transparencies using photolithography of SU-8 photoresist (Microchem) on silicon wafers according to manufacturer protocol. PDMS (Dow Corning) was spin coated at 300 rcf for 60 seconds on these silicon/SU-8 masters and cured at 65°C for 3 hours. Oil reservoirs were created for the device inlets and outlets by punching through-holes in additional PDMS blocks of approximately 10 mm thickness. Glass slides were cleaned by boiling one hour in 1:1:1 ammonium hydroxide: 35% hydrogen peroxide: MilliQ water, then rinsed with water followed by ethanol, dried under nitrogen, and spin coated with PDMS at 4000 rcf for 60 seconds. Final device construction occurred in two oxygen plasma bonding steps; first the master replica was bonded to the spin coated glass slide, and then the inlet/outlet reservoirs and coverglass vapor barrier were bonded. Devices were baked in a 115°C oven for 24–72 hours, then used immediately or stored at room temperature for up to two weeks.

In this design, well dimensions are 400 μm x 200 μm x 100 μm, for an individual well capacity of 8 nL. Oil drainage channels (10μm x 25μm) connect the top of each chamber to the main channel. A main channel 100 μm wide and 25 μm tall bifurcates from an inlet reservoir for 16 channels in parallel, each channel addressing 64 wells. Each device contained 3 arrays and was bonded to a 75mm x 50 mm microscope slide.

Devices were primed with an oil mixture prior to sample loading. This mixture was composed of 0.006% Abil WE 09 (Evonik), 93% Tegosoft DEC (Evonik), and 7% light mineral oil (Sigma part no. M8410). Oil mixtures were used for up to one week before being disposed. The oil mixture was added to the inlets and outlets of the device such that each reservoir was half-filled. The device was placed in a sealed chamber and vacuum was applied to a pressure of -23 inches of mercury. The device was held under vacuum for 5–30 minutes. The pressure was then released and was inspected under the microscope to ensure no air pockets remained in the device channels or chambers. Infrequently, devices would be found to still contain air after five minutes at atmospheric pressure. These devices were placed under vacuum for an additional five minutes. Any devices containing air at this point were considered defective and not used.

Devices were placed on a cooling block on ice during loading (XT Cooling Core, BioCision BCS-511). The aqueous PCR samples were pipetted directly into the device inlet-reservoir under the surface of the residual oil from device priming. A vacuum gasket was aligned with the device outlet reservoirs and attached using double-sided Kapton tape (Ted Pella part no. 16087–19). Vacuum was applied at a regulated -8 in Hg from a bench-top diaphragm vacuum pump, pulling the sample through the device and digitizing the sample. When no remaining aqueous sample was visible in the device inlet, after approximately 8 minutes, remaining oil in the inlet was exchanged for a mixture of 99.97% 50 cSt Silicone oil and 0.03% Abil. This mixture was allowed to flow into the device for 4 minutes before vacuum was released. Any aqueous sample in the device outlet was removed by pipet. Oil reservoirs were filled with the silicone oil and Abil mix.

### Imaging platforms and analysis

After loading, but prior to thermal cycling, arrays were imaged using the Olympus MVX10 macro zoom microscope to generate images of cell nuclei in wells for downstream cell count analysis. The microscope was outfitted with a 2X objective and a 0.63X demagnifying camera adapter, and was used at zoom 1.25X, for a total image magnification of 1.6X. A Hamamatsu Orca R2 camera and automated stage (Prior OptiScan III) both controlled by Nikon Elements BR were used to capture twelve images per array. For fluorescence images, illumination was provided by an X-Cite LED light source (Excelitas Technologies) using filter set for FITC (Semrock) at 100ms exposure. Devices were imaged post-PCR using a Typhoon Trio (GE) at 25μm resolution. Channel PMT voltages were 500V for FAM and 600V for both HEX and Cy5. Typhoon and Olympus image analysis was performed with the open source software ImageJ (https://imagej.nih.gov/ij/) and R (www.r-project.org). A link to custom ImageJ macros and R scripts used to analyze images is available through GitHub (https://github.com/FredHutch/SDGenotypingAnalysis), and contains the executable scripts and images used during the generation of the data presented in Results.

Aqueous area and fluorescence intensity thresholds were used to identify filled vs. non-filled and PCR amplification positive vs. negative wells. Only data from those wells that had an aqueous area at least half of the maximum area were used for further analysis. Fluorescence thresholds were determined using wild-type, mutant, and no template control samples in which the zygosity and PCR-positivity were known a priori, the calculations for which are described in detail in the following paragraphs. For images obtained using the Olympus scope prior to thermal cycling, a threshold was applied above the measured background, the ImageJ particle finder function was used to determine the position of EvaGreen stained cells meeting size and shape criteria within each well boundary after the background regions were identified with the threshold. Large and non-circular particles were counted as two cells. Images were manually inspected to check the accuracy of cell counts as determined by the script. An erroneous cell count value was discovered in ~1% of wells and that value was corrected. PCR status and cell imaging data were matched to determine zygosities in wells with only single cells.

Fluorescence intensity thresholds for calling wells PCR positive or negative were established based on data from arrays containing wild-type or mutant plasmids or no template controls. These thresholds were then applied to heterozygous plasmids and whole-cell samples to prevent bias during analysis. Thresholds were drawn at 3 (amplification probe), 5 (wild-type probe), and 6 (mutant probe) standard deviations above the measured mean intensity of the negative wells. To minimize this error from weakly-positive well intensities, this standard deviation was calculated only for arrays for which the template was off-target for that probe. By this method, no template control and wild type plasmid arrays were used to calculate the negative well standard deviation of the mutant probe, no template control and mutant plasmids were used to calculate the standard deviation for the wild-type probe, and no template control arrays were used to calculate the standard deviation for the amplification probe. A total of 4–5 arrays of data were used to calculate standard deviations (SD’s) for each probe. We calculated the standard deviation by drawing a density curve of the well intensities and measuring the full-width at half-maximum. We then used the formula for standard deviation for a normal distribution, SD = FWHM/2.355. From the 4–5 arrays of data for each probe, we used the maximum standard deviation for thresholding all the subsequent data for that probe.

We noticed small amounts of HEX fluorescence in wells with only FAM and Cy5 positive signal. For example, for runs containing wild-type plasmids (for which HEX fluorescence should be negative), a small amount of HEX fluorescence would occur in FAM+, Cy5+ wells. The amount of HEX signal was much smaller than HEX+ signal in mutant template arrays. This artifact made thresholding in the HEX channel challenging because the distribution of HEX- wells was broad (S7 Fig). We found a positive correlation between FAM signal and HEX signal in these arrays without HEX-specific template. From 5 arrays of control plasmids, we determined a HEX correction factor from the best-fit line of FAM vs. HEX well intensities in wells with templates non-specific to HEX probes. The average best-fit slope was 0.46 I_(FAM)_/I_(HEX)_. Corrected HEX intensities were calculated for all arrays as I_(HEX, corrected)_ = I_(HEX, initial)_—slope*I_(FAM)_. S7 Fig shows control template data before and after this correction.

We also performed an analysis to determine if our thresholds were drawn inappropriately high for one or both allele-specific probes. For appropriately drawn fluorescence thresholds across all three probe channels, we expect that all wells positive for amplification-specific probe fluorescence should also carry positive fluorescence for one or more allele-specific probe. We quantified events where wells were found to be amplification probe positive but allele-specific probe negative. Thresholds unbiased towards one of the allele-specific probes would have similarly low rates of these events. In arrays with wild-type template, 1% of wells were found to be positive for amplification probe signal but negative wild-type probe signal (8/891 wells). In arrays with mutant template, 3% of wells were positive for amplification probe signal and negative for mutant probe signal (19/628 wells). In both cases, many wells were found to be negative for all three probes (1245 in mutant template arrays and 1305 in wild-type template arrays). We concluded that our method of drawing thresholds did not generate meaningful bias towards identification of one specific allele for our assay.

### Allele dropout and failure rates

The thresholds determined from wild-type and mutant plasmids and no template controls were applied to arrays containing a digitized dilution of heterozygous plasmids containing one of each allele. These plasmids were used to assess allele dropout, which for each allele was defined as the number of calls of where that allele was not detected divided by the total number of wells called wild-type, heterozygous, or mutant. Thus, the mutant allele dropout rate (ADO_MUT_) was equal to the number of heterozygous plasmids classified as wild-type divided by the total number of plasmids genotyped, and the wild-type allele dropout rate (ADO_WT_) was equal to the number of heterozygous plasmids classified as mutant divided by the total number of plasmids genotyped.

To make the most conservative calculation of ADO rates, data from wells containing only one copy of plasmid was included, by assuming that wells containing more than one heterozygous plasmid would always be heterozygous. Using the Poisson probability mass function (Eq 1) and the number of negative wells in the arrays, we calculated the expected number of wells containing more than one plasmid, subtracted these from our heterozygous well total, and used the result as the denominator for our ADO calculations. In our ADO and cell occupancy calculations (Fig 4D), we used the fraction of PCR negative wells in an array as the probability that the number of events in that well volume, k, equaled zero (Eq 2) to determine the experimentally observed concentration of events in the original volume (λ). Using this experimentally determined λ we calculated the expected single and multi-event containing wells using Eqs 3 and 4 respectively, for each array.

$$P\left(x=k\;events\right)\;=\;\frac{\lambda^{k}e^{-\lambda }}{k!}$$ (Eq 1)

$$P(x=0\;events)=\frac{Count\;of\;PCR\;negativewells}{Total\;well\;count}=e^{-\lambda }$$ (Eq 2)

$$P(x=1\;event)=\frac{Count\;of\;single\;plasmid\;or\;cell\;wells}{Total\;well\;count}=\lambda e^{-\lambda }$$ (Eq 3)

$$P\left(x>1\;event\right)=\;\frac{Count\;multiple\;plasmid}{Total\;well\;count}\;=1-P\left(x=0\;events\right)\;–\;P\left(x=1\;event\right)$$ (Eq 4)

## Supporting information

S1 Fig Comparison of PCR product generation and yield using various thermal profiles. On-chip ramp rates are slower than conventional thermalcycler rates due to increased thermal mass. Additionally, Mercier et al. (ref 28) found that a modified hot start could increase PCR yield from whole cells. To determine whether these factors impact product generation and yield in bulk PCR, we ran three thermalcycling conditions with the following templates: A: extracted OCI-AML3 DNA 2 ng/rxn, B: whole OCI-AML3 cells 2x10^3^/rxn, C: PBS, and D: water. Reactions were stopped after 33 cycles and products were run on 2% agarose gel. The standard thermal profile consisted of 95°C for 9 min, then 33 cycles of 95°C 15 sec, 60°C 30 sec, and 72°C 30 sec with default ramp rates; the on-chip thermal profile consisted of three cycles of 95°C for 3 minutes and 60°C for 1 minute, then by 30 cycles of 95°C for 15s, and 60°C for 45s with ramp rates of +1.5°C/s and -0.9°C/s; and the standard hot start, slow ramp profile consisted of 95°C for 9 minutes followed by 33 cycles of 95°C for 15 seconds and 60°C for 45 seconds with ramp rates of +1.5°C/s and -0.9°C/s. Ladder bands 100–500 at 100 bp increments are shown. Expected products are at 200 bp (wild-type) and 204 bp (mutant). These results show that using the on-chip thermal profile with slower ramp rates and modified hot start we do get the intended target in bulk-scale PCR. As a bulk PCR cannot directly replicate conditions in a microfluidic well, validation of probe specificity and negative controls were carried out on the microfluidic chip.
(PDF)

S2 Fig Effects of EvaGreen intercalating dye on probe specificity and endpoint fluorescence intensity. Because the SD chip genotyping method used 0.5X EvaGreen for cell-staining, we tested the contribution of this dye to endpoint fluorescence in the FAM channel using standard 10 μL PCR with various templates with and without the FAM probe. Scatter plots of HEX channel (mutant probe) endpoint fluorescence vs. FAM channel (amplification control probe and EvaGreen) endpoint fluorescence in bulk PCR are shown. Compared to samples without FAM probe (only EvaGreen), the change in endpoint signal between positive and negative samples from reactions with both FAM probe and EvaGreen were 1.4 times higher on average. Given this results, we were confident that strongly positive FAM signals would be coming primarily from the FAM probe. This ensures that the FAM signal in the well is coming from amplification specific to the gene of interest and not non-specific products.
(PDF)

S3 Fig Effects of various Triton X-100 concentrations on yield and specificity in bulk-scale PCR. To optimize the endpoint probe signal form cells, we tested the effects of three concentrations of Triton X-100 additive (0%, 0.01%, 0.02%, and 0.05%) on endpoint fluorescence intensity in standard 10 μL PCR. Endpoint fluorescence from mutant and wild-type plasmid templates indicate no change in probe specificity for the three conditions. For samples with OCI-AML3 cells (HET CELLS), we observed no obvious change in the amount of fluorescent signal with increasing Triton X-100 concentration. A decrease in endpoint fluorescence signal for plasmid templates was seen at 0.05%.
(PDF)

S4 Fig Effects of PCR surfactant additives on cell and nuclear membrane integrity determined by fluorescence microscopy. To test the effects of various buffer additives on cell membranes, we observed cells using both a cytoplasm stain and a nuclear stain. We stained cells with calcein violet AM, a cytoplasm stain that is only fluorescent upon enzymatic cleavage in live cells. Because the dye is located in the cytoplasm, cells stained with calcein AM become non-fluorescent upon cell membrane lysis. As a nuclear stain we used EvaGreen, which only stains cells with compromised cell membranes. Calcein signal is preserved in the cells in all the buffers tested. EvaGreen stains cells in PCR buffer with 0.02% and 0.05% Triton X-100, indicating cell death but an intact nucleus. Scale bar is 50μm. No change was seen in cell or nucleus integrity after 30 minute incubation (data not shown). Cell movement may have occurred during filter switching.
(PDF)

S5 Fig SD chip single-cell genotyping quality control well counts for various PCR additive conditions. The SD chip single-cell genotyping method was used with various surfactant concentrations to determine the effect of these additives on the observed frequency of false positives and false negatives in an array. Arrays were loaded with OCI-AML3 cells in one of five buffer conditions: the base PCR buffer as reported in the main text without Triton X-100, buffer with addition of Triton X-100 at 0.01%, 0.02%, or 0.05%, and the base buffer with 0.05% Tween 20 but no Triton X-100. Colored bars represent the fraction of filled wells that fall into each of the four QC categories based on cell imaging data and PCR endpoint fluorescence results (true positive, false positive, false negative, true negative). For each surfactant condition, the fraction of analyzed wells reported is the average across N arrays of that surfactant type (No Surfactant N = 2, 0.05% Tween 20 N = 3, 0.01% Triton X-100 N = 3, 0.02% Triton X-100 N = 2, 0.05% Triton X-100 N = 2). For mixes with 0.02% or 0.05% Triton, 0.5X EvaGreen was added to the PCR buffer for use as a cell stain. In all other conditions, the live-cell stain Vybrant Green was used to stain the cells before adding them to the PCR mix. For Vybrant staining, cells were pelleted and resuspended in 1X PBS containing 5 μM Vybrant Green and incubated at room temperature for 7 minutes. Cells were pelleted again and resuspended in 1X PBS. The proportions of oils for SD chip priming varied with the surfactant content of the aqueous sample. For aqueous samples without added Triton X-100 or Tween 20, a mixture of 0.030% Abil, 93% Tegosoft, and 7.0% light mineral oil was used. For aqueous samples including Triton X-100, the ratio was 0.006% Abil, 93% Tegosoft, and 7% light mineral oil. SD chips were incubated on an Eppendorf Mastercycler with in situ adapter and imaged on a Typhoon FLA 9000. Since instrumentation differed from that used in the main text, single-cell genotyping data from 0.02% Triton X-100 buffer was not included in the main text single-cell genotyping data.
(PDF)

S6 Fig False negative and false positive rates. Well count results from experiments reported in S5 Fig are here reported as false negative and false positive rates, reported as proportions for each array. For each buffer condition, N = 2 or 3 arrays, with a false positive rate (green) and a false negative rate (orange) for each array. A dramatic decrease in the false negative rate is seen in buffers containing 0.02% or 0.05% Triton X-100 compared to other tested conditions. We found that false-positive and false-negative rates were dependent on the concentration of surfactant additives in the PCR buffer. Low amounts of surfactant produced a high number of false negatives and low false positives, indicating incomplete cell lysis; while high amounts of surfactant resulted the majority of cells producing PCR data. Of the conditions tested, buffers containing 0.02% and 0.05% Triton X-100 proved to be the most optimal for reducing both false positives and false negative rates. In bulk reactions, buffer containing 0.05% Triton X-100 decreased endpoint fluorescence (S3 Fig), and thus buffer containing 0.02% Triton X-100 was selected as the optimal buffer for SD chip single-cell genotyping experiments.
(PDF)

S7 Fig Comparison of well intensity distributions before and after HEX bleed-through correction. Each facet represents a single arrays with its template input listed at the top and each point represents a single well of the array. Fluorescence intensity in wells before (Panel A) and after (Panel B) bleed-through correction in the HEX channel. In wild-type samples, HEX channel correction results in a tighter distribution of mutant probe well intensities. Post-HEX correction, fewer false-mutants were found in wild-type plasmid samples. The correction has little effect on mutant and no-template control (NTC) arrays.
(PDF)

S1 Table Primer and allele-specific probe sequences. Nucleotides are listed 5’ to 3’. Locked nucleic acid (LNA) bases are indicated with a “+” before the base. Primers and probes were ordered from IDT DNA. Probes were purified by HPLC. In this table, “FLUOR” designates either HEX or Cy5, as both combinations were used in this manuscript. “Q” designates 3’ Iowa Black® FQ for FAM- and HEX-labeled probes or 3’ Iowa Black® RQ for Cy5-labeled probes.
(PDF)

S1 Text Alternate probe scheme results. (PDF)
