# Supplementary material for: Self-digitization chip for single-cell genotyping of cancer-related mutations
Source: PLoS One. 2018 May 2;13(5):e0196801. doi: 10.1371/journal.pone.0196801 (PMC5931502; doi:10.1371/journal.pone.0196801)
Supplement: S1 Fig — On-chip ramp rates are slower than conventional thermalcycler rates due to increased thermal mass. Additionally, Mercier et al. (ref 28) found that a modified hot start could increase PCR yield from whole cells. To determine whether these factors impact product generation and yield in bulk PCR, we ran three thermalcycling conditions with the following templates: A: extracted OCI-AML3 DNA 2 ng/rxn, B: whole OCI-AML3 cells 2x10^3^/rxn, C: PBS, and D: water. Reactions were stopped after 33 cycles and products were run on 2% agarose gel. The standard thermal profile consisted of 95°C for 9 min, then 33 cycles of 95°C 15 sec, 60°C 30 sec, and 72°C 30 sec with default ramp rates; the on-chip thermal profile consisted of three cycles of 95°C for 3 minutes and 60°C for 1 minute, then by 30 cycles of 95°C for 15s, and 60°C for 45s with ramp rates of +1.5°C/s and -0.9°C/s; and the standard hot start, slow ramp profile consisted of 95°C for 9 minutes followed by 33 cycles of 95°C for 15 seconds and 60°C for 45 seconds with ramp rates of +1.5°C/s and -0.9°C/s. Ladder bands 100–500 at 100 bp increments are shown. Expected products are at 200 bp (wild-type) and 204 bp (mutant). These results show that using the on-chip thermal profile with slower ramp rates and modified hot start we do get the intended target in bulk-scale PCR. As a bulk PCR cannot directly replicate conditions in a microfluidic well, validation of probe specificity and negative controls were carried out on the microfluidic chip. (PDF) [file pone.0196801.s001.pdf]

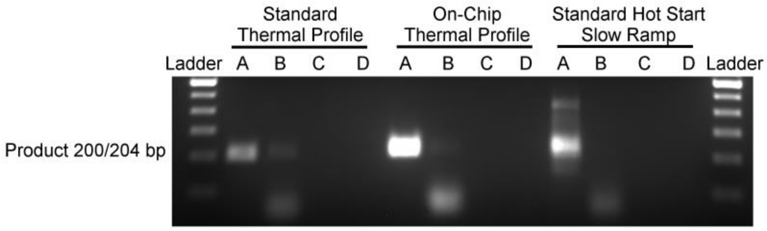

S1 Fig. Comparison of PCR product generation and yield using various thermal profiles. On-chip ramp rates are slower than conventional thermalcycler rates due to increased thermal mass. Additionally, Mercier et al. (ref 28) found that a modified hot start could increase PCR yield from whole cells. To determine whether these factors impact product generation and yield in bulk PCR, we ran three thermalcycling conditions with the following templates: A: extracted OCI-AML3 DNA 2 ng/rxn, B: whole OCI-AML3 cells $2x10^3$/rxn, C: PBS, and D: water. Reactions were stopped after 33 cycles and products were run on 2% agarose gel. The standard thermal profile consisted of 95°C for 9 min, then 33 cycles of 95°C 15 sec, 60°C 30 sec, and 72°C 30 sec with default ramp rates; the on-chip thermal profile consisted of three cycles of 95°C for 3 minutes and 60°C for 1 minute, then by 30 cycles of 95°C for 15s, and 60°C for 45s with ramp rates of +1.5°C/s and -0.9°C/s; and the standard hot start, slow ramp profile consisted of 95°C for 9 minutes followed by 33 cycles of 95°C for 15 seconds and 60°C for 45 seconds with ramp rates of +1.5°C/s and -0.9°C/s. Ladder bands 100-500 at 100 bp increments are shown. Expected products are at 200 bp (wild-type) and 204 bp (mutant). These results show that using the on-chip thermal profile with slower ramp rates and modified hot start we do get the intended target in bulk-scale PCR. As a bulk PCR cannot directly replicate conditions in a microfluidic well, validation of probe specificity and negative controls were carried out on the microfluidic chip.
