# Supplementary material for: Self-digitization chip for single-cell genotyping of cancer-related mutations
Source: PLoS One. 2018 May 2;13(5):e0196801. doi: 10.1371/journal.pone.0196801 (PMC5931502; doi:10.1371/journal.pone.0196801)
Supplement: S2 Fig — Because the SD chip genotyping method used 0.5X EvaGreen for cell-staining, we tested the contribution of this dye to endpoint fluorescence in the FAM channel using standard 10 μL PCR with various templates with and without the FAM probe. Scatter plots of HEX channel (mutant probe) endpoint fluorescence vs. FAM channel (amplification control probe and EvaGreen) endpoint fluorescence in bulk PCR are shown. Compared to samples without FAM probe (only EvaGreen), the change in endpoint signal between positive and negative samples from reactions with both FAM probe and EvaGreen were 1.4 times higher on average. Given this results, we were confident that strongly positive FAM signals would be coming primarily from the FAM probe. This ensures that the FAM signal in the well is coming from amplification specific to the gene of interest and not non-specific products. (PDF) [file pone.0196801.s002.pdf]

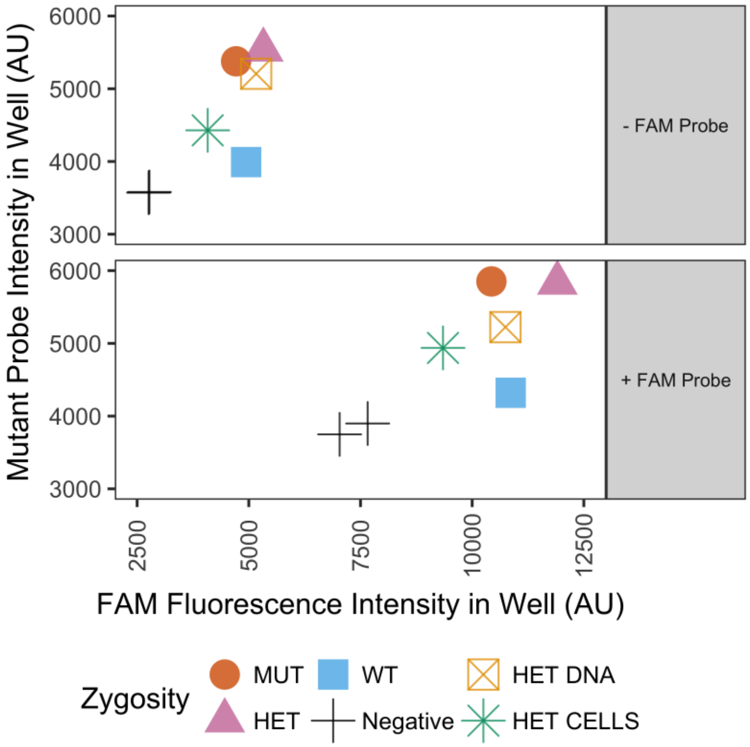

S2 Fig. Effects of EvaGreen intercalating dye on probe specificity and endpoint fluorescence intensity. Because the SD chip genotyping method used 0.5X EvaGreen for cell-staining, we tested the contribution of this dye to endpoint fluorescence in the FAM channel using standard 10 μL PCR with various templates with and without the FAM probe. Scatter plots of HEX channel (mutant probe) endpoint fluorescence vs. FAM channel (amplification control probe and EvaGreen) endpoint fluorescence in bulk PCR are shown. Compared to samples without FAM probe (only EvaGreen), the change in endpoint signal between positive and negative samples from reactions with both FAM probe and EvaGreen were 1.4 times higher on average. Given this results, we were confident that strongly positive FAM signals would be coming primarily from the FAM probe. This ensures that the FAM signal in the well is coming from amplification specific to the gene of interest and not non-specific products.
