# Supplementary material for: Self-digitization chip for single-cell genotyping of cancer-related mutations
Source: PLoS One. 2018 May 2;13(5):e0196801. doi: 10.1371/journal.pone.0196801 (PMC5931502; doi:10.1371/journal.pone.0196801)
Supplement: S3 Fig — To optimize the endpoint probe signal form cells, we tested the effects of three concentrations of Triton X-100 additive (0%, 0.01%, 0.02%, and 0.05%) on endpoint fluorescence intensity in standard 10 μL PCR. Endpoint fluorescence from mutant and wild-type plasmid templates indicate no change in probe specificity for the three conditions. For samples with OCI-AML3 cells (HET CELLS), we observed no obvious change in the amount of fluorescent signal with increasing Triton X-100 concentration. A decrease in endpoint fluorescence signal for plasmid templates was seen at 0.05%. (PDF) [file pone.0196801.s003.pdf]

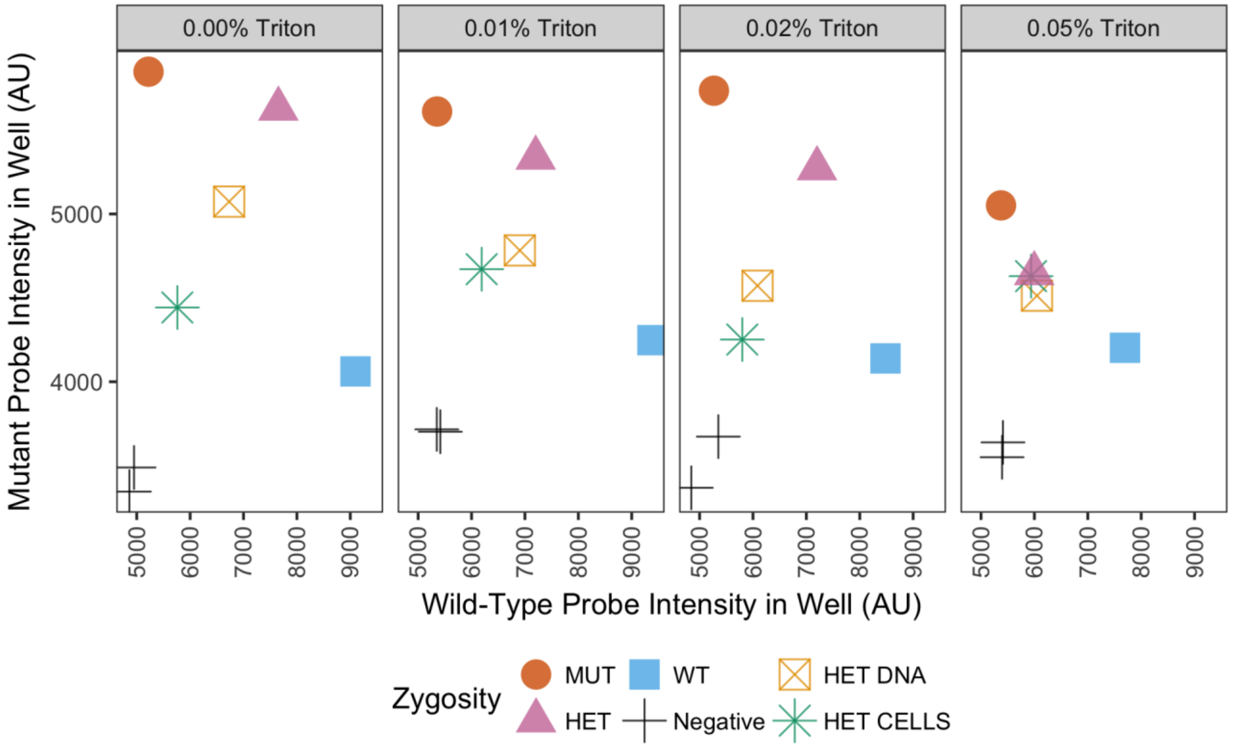

S3 Fig. Effects of various Triton X-100 concentrations on yield and specificity in bulk-scale PCR. To optimize the endpoint probe signal form cells, we tested the effects of three concentrations of Triton X-100 additive (0%, 0.01%, 0.02%, and 0.05%) on endpoint fluorescence intensity in standard 10 μL PCR. Endpoint fluorescence from mutant and wild-type plasmid templates indicate no change in probe specificity for the three conditions. For samples with OCI-AML3 cells (HET CELLS), we observed no obvious change in the amount of fluorescent signal with increasing Triton X-100 concentration. A decrease in endpoint fluorescence signal for plasmid templates was seen at 0.05%.
