# Supplementary material for: Self-digitization chip for single-cell genotyping of cancer-related mutations
Source: PLoS One. 2018 May 2;13(5):e0196801. doi: 10.1371/journal.pone.0196801 (PMC5931502; doi:10.1371/journal.pone.0196801)
Supplement: S4 Fig — To test the effects of various buffer additives on cell membranes, we observed cells using both a cytoplasm stain and a nuclear stain. We stained cells with calcein violet AM, a cytoplasm stain that is only fluorescent upon enzymatic cleavage in live cells. Because the dye is located in the cytoplasm, cells stained with calcein AM become non-fluorescent upon cell membrane lysis. As a nuclear stain we used EvaGreen, which only stains cells with compromised cell membranes. Calcein signal is preserved in the cells in all the buffers tested. EvaGreen stains cells in PCR buffer with 0.02% and 0.05% Triton X-100, indicating cell death but an intact nucleus. Scale bar is 50μm. No change was seen in cell or nucleus integrity after 30 minute incubation (data not shown). Cell movement may have occurred during filter switching. (PDF) [file pone.0196801.s004.pdf]

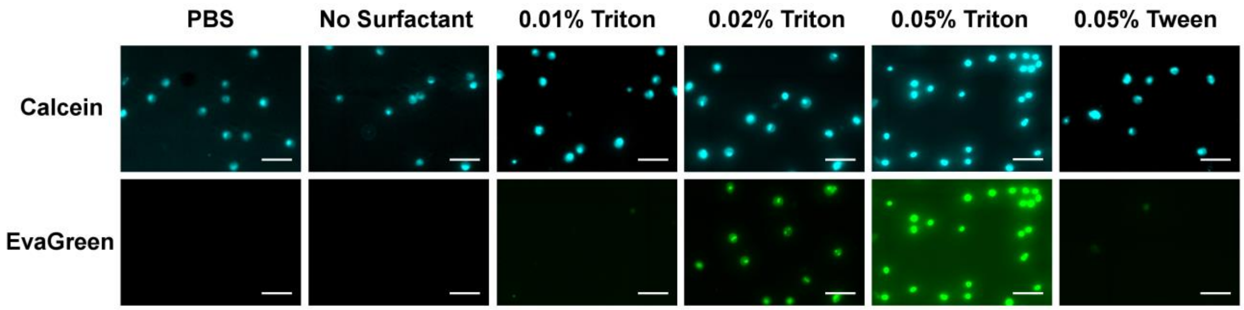

S4 Fig. Effects of PCR surfactant additives on cell and nuclear membrane integrity determined by fluorescence microscopy. To test the effects of various buffer additives on cell membranes, we observed cells using both a cytoplasm stain and a nuclear stain. We stained cells with calcein violet AM, a cytoplasm stain that is only fluorescent upon enzymatic cleavage in live cells. Because the dye is located in the cytoplasm, cells stained with calcein AM become non-fluorescent upon cell membrane lysis. As a nuclear stain we used EvaGreen, which only stains cells with compromised cell membranes. Calcein signal is preserved in the cells in all the buffers tested. EvaGreen stains cells in PCR buffer with 0.02% and 0.05% Triton X-100, indicating cell death but an intact nucleus. Scale bar is 50µm. No change was seen in cell or nucleus integrity after 30 minute incubation (data not shown). Cell movement may have occurred during filter switching.
