# Supplementary material for: Self-digitization chip for single-cell genotyping of cancer-related mutations
Source: PLoS One. 2018 May 2;13(5):e0196801. doi: 10.1371/journal.pone.0196801 (PMC5931502; doi:10.1371/journal.pone.0196801)
Supplement: S5 Fig — The SD chip single-cell genotyping method was used with various surfactant concentrations to determine the effect of these additives on the observed frequency of false positives and false negatives in an array. Arrays were loaded with OCI-AML3 cells in one of five buffer conditions: the base PCR buffer as reported in the main text without Triton X-100, buffer with addition of Triton X-100 at 0.01%, 0.02%, or 0.05%, and the base buffer with 0.05% Tween 20 but no Triton X-100. Colored bars represent the fraction of filled wells that fall into each of the four QC categories based on cell imaging data and PCR endpoint fluorescence results (true positive, false positive, false negative, true negative). For each surfactant condition, the fraction of analyzed wells reported is the average across N arrays of that surfactant type (No Surfactant N = 2, 0.05% Tween 20 N = 3, 0.01% Triton X-100 N = 3, 0.02% Triton X-100 N = 2, 0.05% Triton X-100 N = 2). For mixes with 0.02% or 0.05% Triton, 0.5X EvaGreen was added to the PCR buffer for use as a cell stain. In all other conditions, the live-cell stain Vybrant Green was used to stain the cells before adding them to the PCR mix. For Vybrant staining, cells were pelleted and resuspended in 1X PBS containing 5 μM Vybrant Green and incubated at room temperature for 7 minutes. Cells were pelleted again and resuspended in 1X PBS. The proportions of oils for SD chip priming varied with the surfactant content of the aqueous sample. For aqueous samples without added Triton X-100 or Tween 20, a mixture of 0.030% Abil, 93% Tegosoft, and 7.0% light mineral oil was used. For aqueous samples including Triton X-100, the ratio was 0.006% Abil, 93% Tegosoft, and 7% light mineral oil. SD chips were incubated on an Eppendorf Mastercycler with in situ adapter and imaged on a Typhoon FLA 9000. Since instrumentation differed from that used in the main text, single-cell genotyping data from 0.02% Triton X-100 buffer was not included in the main text si [file pone.0196801.s005.pdf]

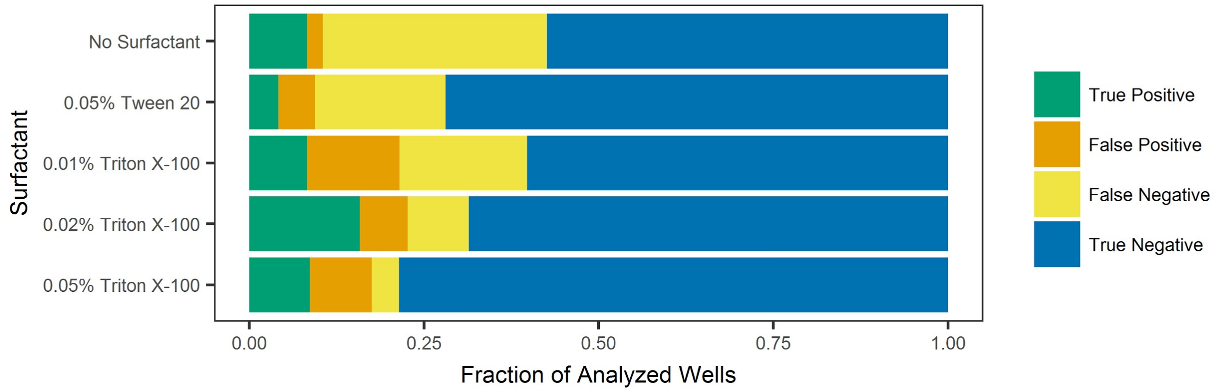

S5 Fig. SD chip single-cell genotyping quality control well counts for various PCR additive conditions. The SD chip single-cell genotyping method was used with various surfactant concentrations to determine the effect of these additives on the observed frequency of false positives and false negatives in an array. Arrays were loaded with OCI-AML3 cells in one of five buffer conditions: the base PCR buffer as reported in the main text without Triton X-100, buffer with addition of Triton X-100 at 0.01%, 0.02%, or 0.05%, and the base buffer with 0.05% Tween 20 but no Triton X-100. Colored bars represent the fraction of filled wells that fall into each of the four QC categories based on cell imaging data and PCR endpoint fluorescence results (true positive, false positive, false negative, true negative). For each surfactant condition, the fraction of analyzed wells reported is the average across N arrays of that surfactant type (No Surfactant N = 2, 0.05% Tween 20 N = 3, 0.01% Triton X-100 N = 3, 0.02% Triton X-100 N = 2, 0.05% Triton X-100 N = 2). For mixes with 0.02% or 0.05% Triton, 0.5X EvaGreen was added to the PCR buffer for use as a cell stain. In all other conditions, the live-cell stain Vybrant Green was used to stain the cells before adding them to the PCR mix. For Vybrant staining, cells were pelleted and resuspended in 1X PBS containing 5 µM Vybrant Green and incubated at room temperature for 7 minutes. Cells were pelleted again and resuspended in 1X PBS. The proportions of oils for SD chip priming varied with the surfactant content of the aqueous sample. For aqueous samples without added Triton X-100 or Tween 20, a mixture of 0.030% Abil, 93% Tegosoft, and 7.0% light mineral oil was used. For aqueous samples including Triton X-100, the ratio was 0.006% Abil, 93% Tegosoft, and 7% light mineral oil. SD chips were incubated on an Eppendorf Mastercycler with in situ adapter and imaged on a Typhoon FLA 9000. Since instrumentation differed from that used in the main text, single-cell genotyping data from 0.02% Triton X-100 buffer was not included in the main text single-cell genotyping data.
