# Supplementary material for: Self-digitization chip for single-cell genotyping of cancer-related mutations
Source: PLoS One. 2018 May 2;13(5):e0196801. doi: 10.1371/journal.pone.0196801 (PMC5931502; doi:10.1371/journal.pone.0196801)
Supplement: S6 Fig — Well count results from experiments reported in S5 Fig are here reported as false negative and false positive rates, reported as proportions for each array. For each buffer condition, N = 2 or 3 arrays, with a false positive rate (green) and a false negative rate (orange) for each array. A dramatic decrease in the false negative rate is seen in buffers containing 0.02% or 0.05% Triton X-100 compared to other tested conditions. We found that false-positive and false-negative rates were dependent on the concentration of surfactant additives in the PCR buffer. Low amounts of surfactant produced a high number of false negatives and low false positives, indicating incomplete cell lysis; while high amounts of surfactant resulted the majority of cells producing PCR data. Of the conditions tested, buffers containing 0.02% and 0.05% Triton X-100 proved to be the most optimal for reducing both false positives and false negative rates. In bulk reactions, buffer containing 0.05% Triton X-100 decreased endpoint fluorescence (S3 Fig), and thus buffer containing 0.02% Triton X-100 was selected as the optimal buffer for SD chip single-cell genotyping experiments. (PDF) [file pone.0196801.s006.pdf]

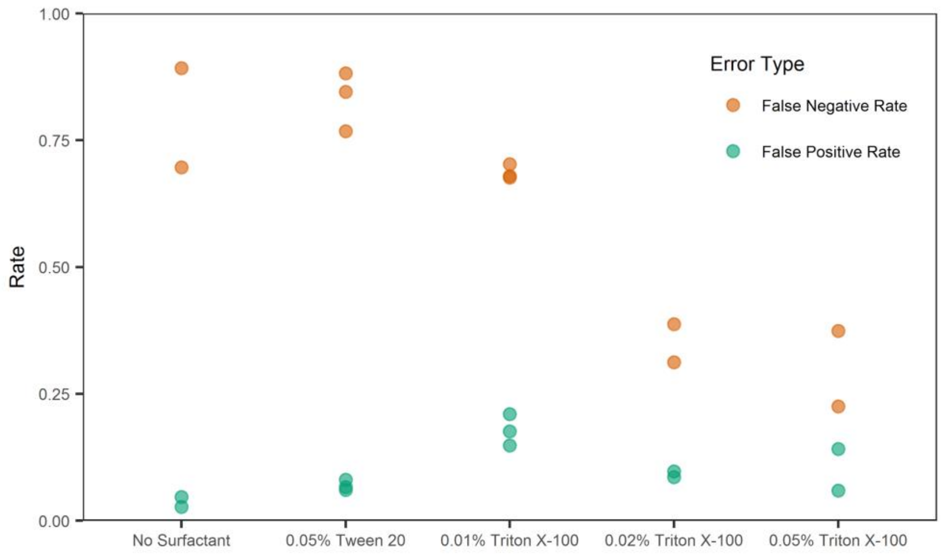

S6 Fig. False negative and false positive rates. Well count results from experiments reported in S5 Fig are here reported as false negative and false positive rates, reported as proportions for each array. For each buffer condition, N = 2 or 3 arrays, with a false positive rate (green) and a false negative rate (orange) for each array. A dramatic decrease in the false negative rate is seen in buffers containing 0.02% or 0.05% Triton X-100 compared to other tested conditions. We found that false-positive and false-negative rates were dependent on the concentration of surfactant additives in the PCR buffer. Low amounts of surfactant produced a high number of false negatives and low false positives, indicating incomplete cell lysis; while high amounts of surfactant resulted the majority of cells producing PCR data. Of the conditions tested, buffers containing 0.02% and 0.05% Triton X-100 proved to be the most optimal for reducing both false positives and false negative rates. In bulk reactions, buffer containing 0.05% Triton X-100 decreased endpoint fluorescence (see S3 Fig), and thus buffer containing 0.02% Triton X-100 was selected as the optimal buffer for SD chip single-cell genotyping experiments.
