# Supplementary material for: Self-digitization chip for single-cell genotyping of cancer-related mutations
Source: PLoS One. 2018 May 2;13(5):e0196801. doi: 10.1371/journal.pone.0196801 (PMC5931502; doi:10.1371/journal.pone.0196801)
Supplement: S7 Fig — Each facet represents a single arrays with its template input listed at the top and each point represents a single well of the array. Fluorescence intensity in wells before (Panel A) and after (Panel B) bleed-through correction in the HEX channel. In wild-type samples, HEX channel correction results in a tighter distribution of mutant probe well intensities. Post-HEX correction, fewer false-mutants were found in wild-type plasmid samples. The correction has little effect on mutant and no-template control (NTC) arrays. (PDF) [file pone.0196801.s007.pdf]

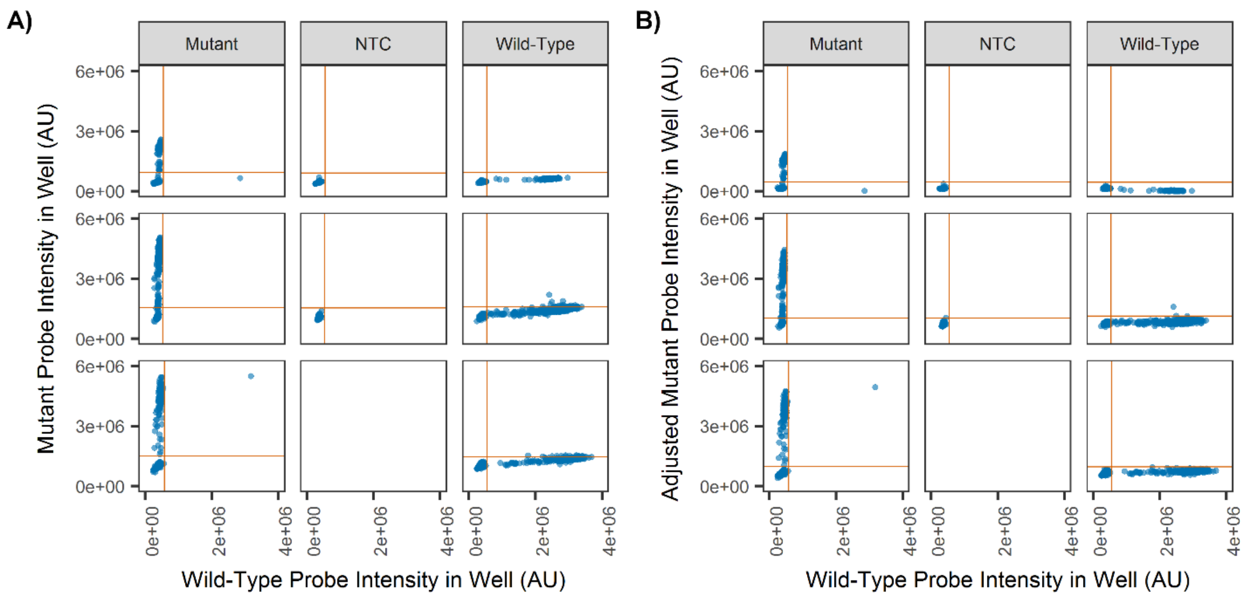

S7 Fig. Comparison of well intensity distributions before and after HEX bleed-through correction. Each facet represents a single arrays with its template input listed at the top and each point represents a single well of the array. Fluorescence intensity in wells before (Panel A) and after (Panel B) bleed-through correction in the HEX channel. In wild-type samples, HEX channel correction results in a tighter distribution of mutant probe well intensities. Post-HEX correction, fewer false-mutants were found in wild-type plasmid samples. The correction has little effect on mutant and no-template control (NTC) arrays.
