# Supplementary material for: Self-digitization chip for single-cell genotyping of cancer-related mutations
Source: PLoS One. 2018 May 2;13(5):e0196801. doi: 10.1371/journal.pone.0196801 (PMC5931502; doi:10.1371/journal.pone.0196801)
Supplement: S1 Table — Nucleotides are listed 5’ to 3’. Locked nucleic acid (LNA) bases are indicated with a “+” before the base. Primers and probes were ordered from IDT DNA. Probes were purified by HPLC. In this table, “FLUOR” designates either HEX or Cy5, as both combinations were used in this manuscript. “Q” designates 3’ Iowa Black® FQ for FAM- and HEX-labeled probes or 3’ Iowa Black® RQ for Cy5-labeled probes. (PDF) [file pone.0196801.s008.pdf]

Forward primer	GTCTATGAAGTGTTGTGGTT
Reverse primer	CGGTAGGGAAAGTTCTCA
Wild-type specific probe	(FLUOR)-AGAT+CT+CT+G+G+CAG-Q
Mutation specific probe	(FLUOR)-AGA+TC+T+C+T+GT+CTG-Q
Amplification control probe	FAM-ATT+TCTGTA+ACAGTT+GATATCTGGC-Q

S1 Table. Primer and allele-specific probe sequences. Nucleotides are listed 5' to 3'. Locked nucleic acid (LNA) bases are indicated with a "+" before the base. Primers and probes were ordered from IDT DNA. Probes were purified by HPLC. In this table, "FLUOR" designates either HEX or Cy5, as both combinations were used in this manuscript. "Q" designates 3' Iowa Black® FQ for FAM- and HEX-labeled probes or 3' Iowa Black® RQ for Cy5-labeled probes.
