# Supplementary material for: Self-digitization chip for single-cell genotyping of cancer-related mutations
Source: PLoS One. 2018 May 2;13(5):e0196801. doi: 10.1371/journal.pone.0196801 (PMC5931502; doi:10.1371/journal.pone.0196801)
Supplement: S1 Text — (PDF) [file pone.0196801.s009.pdf]

S1 Text. Alternate probe scheme results. While the main probe scheme used in this paper was $AMP_{FAM}WT_{Cy5}MUT_{HEX}$, we also validated probes with the fluorophores on the allele-specific probes swapped, $AMP_{FAM}WT_{HEX}MUT_{Cy5}$ scheme. In the $AMP_{FAM}WT_{HEX}MUT_{Cy5}$ probe scheme, a higher error rate was observed with wild-type false allele rate 2% (5 false allele out of N = 278 wild-type positive observations), and mutant 0.4% (1 false allele out of N = 275 mutant positive observations). Additionally, seven amplification positive wells were found in the no template controls for this probe scheme (N = 2 arrays). We also calculated the allele dropout rate for this probe swap scheme, and found a ADO_{WT} of 8.6 ± 2.8 percent and a ADO_{MUT} of 9.4 ± 2.3 percent. (N = 1085 zygosity calls, N = 2 days, error is standard deviation between 6 array replicates.
